# Anti-Diabetic Potential of *Sargassum horneri* and *Ulva australis* Extracts In Vitro and In Vivo

**DOI:** 10.3390/cimb45090473

**Published:** 2023-09-13

**Authors:** Young-Hyeon Lee, Hye-Ran Kim, Min-Ho Yeo, Sung-Chun Kim, Ho-Bong Hyun, Young-Min Ham, Yong-Hwan Jung, Hye-Sook Kim, Kyung-Soo Chang

**Affiliations:** 1 Department of Clinical Laboratory Science, Catholic University of Pusan, Busan 46252, Republic of Korea; dudgus901622@gmail.com (Y.-H.L.); dualsgh136@naver.com (M.-H.Y.); 2 Department of Biomedical Laboratory Science, Dong-Eui Institute of Technology, Busan 47230, Republic of Korea; hrkim@dit.ac.kr; 3 Biodiversity Research Institute, Jeju Technopark, Jeju 63608, Republic of Korea; sckim@jejutp.or.kr (S.-C.K.); hyebong@jejutp.or.kr (H.-B.H.); hijel@jejutp.or.kr (Y.-M.H.); yhjung@jejutp.or.kr (Y.-H.J.); 4 Division of International Infectious Diseases Control, Faculty of Pharmaceutical Sciences, Okayama University, Tsushima-Naka, Kita-Ku, Okayama 700-8530, Japan; hskim@cc.okayama-u.ac.jp

**Keywords:** diabetes, *Sargassum horneri*, *Ulva australis*, extracts

## Abstract

*Sargassum horneri* (SH) and *Ulva australis* (UA) are marine waste resources that cause environmental and economic problems when entering or multiplying the coastal waters of Jeju Island. We analyzed their anti-diabetic efficacy to assess their reusability as functional additives. The alpha-glucosidase inhibitory activity of SH and UA extracts was confirmed, and the effect of UA extract was higher than that of SH. After the induction of insulin-resistant HepG2 cells, the effects of the two marine extracts on oxidative stress, intracellular glucose uptake, and glycogen content were compared to the positive control, metformin. Treatment of insulin-resistant HepG2 cells with SH and UA resulted in a concentration-dependent decrease in oxidative stress and increased intracellular glucose uptake and glycogen content. Moreover, SH and UA treatment upregulated the expression of *IRS-1*, *AKT*, and *GLUT4*, which are suppressed in insulin resistance, to a similar degree to metformin, and suppressed the expression of *FoxO1*, *PEPCK* involved in gluconeogenesis, and *GSK-3β* involved in glycogen metabolism. The oral administration of these extracts to rats with streptozotocin-induced diabetes led to a higher weight gain than that in the diabetic group. Insulin resistance and oral glucose tolerance are alleviated by the regulation of blood glucose. Thus, the SH and UA extracts may be used in the development of therapeutic agents or supplements to improve insulin resistance.

## 1. Introduction

Diabetes mellitus (DM) is a severe and chronic metabolic disease characterized by increased blood glucose levels due to an imbalance in glucose homeostasis caused by reduced insulin secretion, insulin resistance (IR), or a combination of these factors [1,2]. According to the latest report from the International Diabetes Federation (IDF), over 460 million adults worldwide have been diagnosed with diabetes, and this number is expected to increase to approximately 700 million by 2045 [3]. Diabetes is classified into type 1 diabetes, which is caused by a decrease in insulin secretion due to a reduction in β cells in the pancreas, and type 2 diabetes, which is caused by a reduction in insulin secretion and IR [4]. More than 90% of the people worldwide with the illness have type 2 diabetes [5].

Type 2 DM can develop at a younger age because of a high-fat diet, high-calorie diet, a lifestyle with low physical activity, IR, and insufficient insulin metabolism [6]. IR is defined as impaired glucose uptake, metabolism, and storage [7]. Liver, skeletal muscle, and adipose tissues are representative insulin-sensitive tissues that cause hyperglycemia, hyperinsulinemia, and dyslipidemia. As the liver absorbs 34% of orally administered glucose and supplies 90% of the glucose required by the body in a fasting state, there is a growing need for research on glucose absorption and hypoglycemic mechanisms [8,9]. Accordingly, some studies have been conducted to evaluate the antidiabetic efficacy by increasing enzyme activity or enzyme expression in hepatocytes whose activity is reduced due to diabetes [10]. In addition, because the expression of genes, such as insulin receptor substrate-1 (*IRS1*), protein kinase B (*AKT*), and *GLUT4*, tends to significantly reduce insulin resistance, gene regulation related to insulin metabolism can be used as an index to evaluate diabetes treatment [11]. Based on this evidence, drugs for treating diabetes are being developed and a combination of exercise and diet is being used to manage diabetes. However, no fundamental treatment has yet been established for this condition. Biguanides, sulfonylureas/glucosidase inhibitors, and oral hypoglycemic agents such as thiazolidinedione and insulin are used to treat diabetes; however, they cause gastrointestinal side effects such as abdominal pain, diarrhea, and nausea [12,13]. To address these side effects, extensive research has been conducted on natural substances that effectively prevent and treat type 2 diabetes.

Various foods with a low glycemic index, including nuts, vegetables, and seaweeds, are known to prevent and improve DM [14,15,16]. Seaweeds are generally categorized into red, brown, and green algae according to their color and are reported to be rich in proteins, minerals, and vitamins, as well as in dietary fiber, a non-starch polysaccharide that digestive enzymes cannot break down [17]. Seaweed is also effective in preventing and managing diabetes because it is low in calories, has a low glycemic index, and is rich in antioxidant nutrients [18]. *Sargassum horneri* (SH), used in the present study, is a delicacy in East Asia, whereas *Ulva australis* (UA) is used as food in Europe. However, SH and UA have been identified as the main causes of coastal pollution on Jeju Island [19,20].

SH farming is promoted in Chinese waters, and fragments of growing SH are scattered all over the southern coast and in Japan, with an average of 1000–9000 tons introduced to Jeju Island [21]. UA is found mainly along the coast, where algal circulation is inactive, and its annual generation is estimated to be 10,000 tons [22]. SH and UA are introduced and generated in coastal waters, causing loss of beach function and foul odors due to decay, adding to the inconvenience of nearby residents and tourists. Climate change is expected to exacerbate this situation [23]. Various studies have been conducted to determine the potential applications of these two seaweeds, which are recognized as organic waste resources. Several studies have highlighted that SH has antitumor [24], anti-inflammatory [25], and anti-allergy effects [26]. Studies of the antihyperlipidemic [27], antiviral [28], polysaccharide structure, and physicochemical properties [29] of UA are currently in progress. Both seaweeds are potentially valuable for blood sugar control; however, research to elucidate their comprehensive efficacy against hepatic glucose metabolism disorders and the associated mechanisms remains scarce. In addition, according to the results of previous studies, neophytadien, hexadecanoic acid, and ethyl ester were identified as major compounds in SH extracts, and 2-Hexadecen-1-ol, 3,7,11,15-tetramethyl-acetate, [R-[R*,R*-(E)]] in UA extracts [30]. There is a need for research on blood sugar control using SH and UA extracts containing compounds with physiological activities such as antibacterial and antioxidant effects.

Therefore, in the present study, the enzyme inhibitory potential of SH and UA extracts was analyzed and their effects on the regulation of glucose metabolism, gluconeogenesis, and *IRS*/*AKT* signaling pathways were compared in insulin-resistant HepG2 cells. In addition, by analyzing whether insulin resistance could be improved by administering the two extracts to STZ-induced diabetic mice, we sought to confirm the possibility of using SH and UA to improve diabetes.

## 2. Materials and Methods

### 2.1. Preparation of SH and UA Ethanol Extracts

SH and UA were collected from the coast of Jeju Island and washed, dried, and powdered before use. The dried samples (50 g) were immersed in 1 L of 80% ethanol and extracted by stirring at room temperature for 24 h. The stirred extracted material was filtered using a 0.45 µm bottle-top vacuum filter (Corning Inc., Corning, NY, USA), and the filtered material was evaporated from ethanol using a rotary concentrator (R100 + B100, Lab Scitech, Corona, CA, USA) and then lyophilized to prepare an extract powder. Sample extraction was performed twice using the same procedure. The prepared samples were stored at −20 °C, suspended in a cell culture medium or sterile distilled water at 10 mg/mL concentration, and diluted before use.

### 2.2. α-Glucosidase Inhibitory Assay

*α*-glucosidase inhibitory activity was analyzed using an *α*-glucosidase inhibitor screening kit (K938-100, BioVision, Milpitas, CA, USA). After diluting the extract, 10 μL was dispensed into a 96-well plate, and 10 μL of *α*-glucosidase enzyme solution was added. The volume of each well was adjusted to 80 μL with assay buffer and incubated for 15 to 20 min at room temperature. Then, *α*-glucosidase assay buffer and *α*-glucosidase substrate mix were mixed, and 20 μL of the solution was added to each well. The absorbance was measured at 410 nm using a microplate reader (Varioskan LUX, Thermo Fisher Scientific, Waltham, MA, USA).

### 2.3. Cell Culture and Induction of Insulin Resistance Model

The HepG2 human liver cancer cell line was purchased from Korean Cell Line Bank (Seoul, Republic of Korea). HepG2 cells were cultured in Minimum Essential Medium Eagle (MEM, Welgene, Seoul, Republic of Korea) medium supplemented with 10% fetal bovine serum (FBS, Welgene) and 1% penicillin–streptomycin (PS, Welgene) at 37 °C and 5% CO_2_ incubator. The cells were cultured to approximately 70–80% confluence, passaged every 2–3 days, and used for the experiments. The glucose concentrations in the MEM were 5 and 30 mM.

The IR model was established based on the results of a previous study [31]. HepG2 cells were seeded in a plate and then cultured in an incubator at 37 °C and 5% CO_2_ for 24 h using a culture medium. To induce IR-HepG2 cells, 1 μM insulin (Sigma-Aldrich, St. Louis, MI, USA) was added to serum-free MEM, and D-glucose was added at a concentration of 30 mM and cultured for 48 h. As a control, normal HepG2 cells were cultured in insulin-free medium containing 5 mM D-glucose. Next, the cells were treated with different concentrations of the extract or metformin (Sigma-Aldrich) at a concentration of 1 mM and cultured for 24 h. Finally, insulin (100 nM) was added, incubated for 30 min, and used for subsequent analysis.

### 2.4. Cell Viability Assay

The effect of the extract on the viability of HepG2 and IR-HepG2 cells was evaluated using the WST-1 reagent (Cellvia, AbFrontier, Seoul, Republic of Korea). HepG2 cells were seeded in 96-well culture plates at 1 × 10^5^ cells/mL and cultured for 24 h. Extracts were then prepared at different concentrations (125, 250, 500, and 1000 μg/mL) in a medium without FBS, treated with cells, and cultured in a 37 °C 5% CO_2_ incubator for 24 h. Next, 10 μL of WST-1 reagent was added to the medium and incubated for 30 min in a 37 °C, 5% CO_2_ incubator. The absorbance was measured at 450 nm using a microplate reader (Varioskan LUX, Thermo Fisher Scientific). Cell viability was compared to the absorbance of the control group.

### 2.5. Reactive Oxygen Species (ROS) Detection Assay

Reactive oxygen species (ROS) were analyzed using 2′,7′-dichlorofluorescein diacetate (DCFH-DA, Sigma, USA). After the cells were dispensed at 1 × 10^5^ cells/mL in a 96-well plate, insulin resistance was induced and the cells were treated with the extract or metformin. After washing the cells twice with phosphate-buffered saline (PBS), 10 μM DCFH-DA was added, and the solution was incubated for 40 min in a 37 °C and 5% CO_2_ incubator. The fluorescence intensity of DCFH-DA was measured at 485 nm (excitation) and 525 nm (reflection) using a microplate reader (Varioskan LUX, Thermo Fisher Scientific).

HepG2 cells were seeded onto a Lab-Tek 4 well chamber slide and treated according to a previously described protocol. The fluorescence intensity was confirmed using a fluorescence microscope (BX51; Olympus Optical Co., Tokyo, Japan).

### 2.6. Cellular Glucose Uptake Assay

Intracellular glucose uptake was analyzed using 2-deoxy-2-[(7-nitro-2,1,3-benzoxadiazol-4-yl)amino]-D-glucose (2-NBDG; Invitrogen, Waltham, MA, USA), as previously described [32]. After preparing the insulin resistance model, the extract and metformin were administered and incubated for 24 h. The cells were then treated with insulin (100 nM) for 30 min, and 2-NBDG (40 μM) was added and incubated for 30 min. Fluorescence was measured after washing the cells three times with cold PBS. The fluorescence intensity of 2-NBDG was measured at 460 nm (excitation) and 528 nm (reflection) using a microplate reader (Varioskan LUX, Thermo Fisher Scientific).

HepG2 cells were seeded onto Lab-Tek 4 well chamber slide and treated according to the same protocol. To confirm the number of cells, the nucleic acid dye Hoechst 33342 (Invitrogen) was added and the cells were incubated for 30 min. After washing with cold PBS, cells were examined under a fluorescence microscope (BX51; Olympus Optical Co.).

### 2.7. Glycogen Content Assay

After inducing insulin resistance in HepG2 cells, the extract and metformin were administered as previously described. HepG2 cells were homogenized in 200 μL of dH_2_O. The homogenate was boiled for 10 min to inactivate the enzymes, centrifuged at 16,000× *g*, and the supernatant was stored on ice. The glycogen content of the supernatant was analyzed using a glycogen colorimetric assay kit (Biovision, Milpitas, CA, USA) according to the manufacturer’s instructions. The glycogen content of the cells was calculated using the total glucose level minus the free glucose background for each sample and normalized to the protein level.

### 2.8. Quantitative RT-PCR Analysis

HepG2 cells were washed with 1 × PBS and total RNA was extracted using an Accuprep^®^ universal RNA extraction kit (Bioneer, Daejeon, Republic of Korea). Extracted RNA was quantified using a NanoDrop 2000 (Thermo Fisher Scientific). Next, 1 μg of RNA was subjected to qRT-PCR on a 7500 Fast Real-Time PCR system (Applied Biosystems, Waltham, MA, USA) using a One-Step TB Green^®^ PrimeScript^™^ RT-PCR Kit (Takara, Japan). The conditions were 42 °C for 50 s, 95 °C for 10 s, followed by 40 cycles of 95 °C for 5 s, 55 or 58 °C for 30 s, and 72 °C for 30 s. The primers used for qRT-PCR analysis are listed in Table 1. The mRNA levels of the target gene in the same sample were normalized to that of *β*-actin, a housekeeping gene. Data were expressed as relative fold change in transcript levels relative to controls using the 2^−ΔΔCT^ equation.

### 2.9. Animal Experiments

**Scheme 1:**
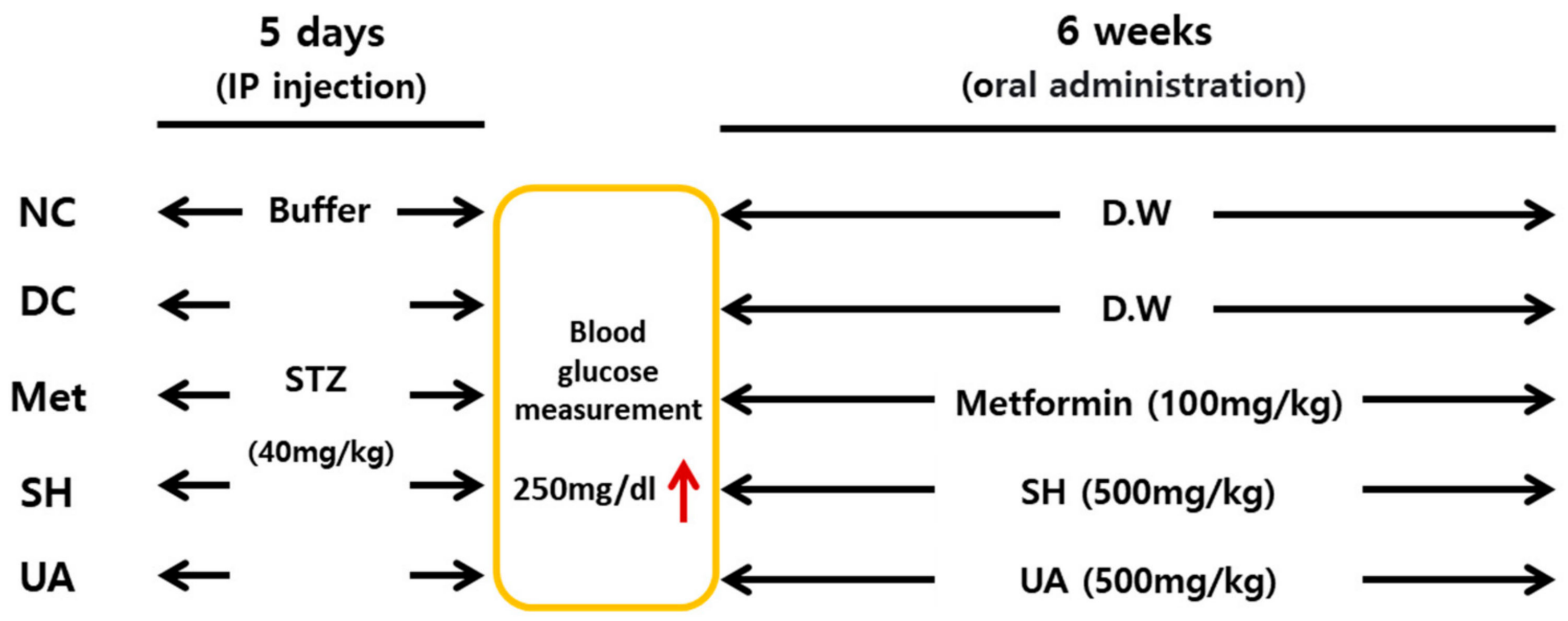
Experimental plan for amelioration of diabetes in streptozotocin-induced diabetic animals by treatment with SH and UA extracts. After 5 days of IP dosing with Streptozotocin, confirm that blood glucose reaches 250 mg/dl or higher. Administer the drug orally for the next 6 weeks. NC, normal control; DC, diabetic control; Met, metformin 100 mg/kg; SH, *Sargassum horneri* extract 500 mg/kg; UA, *Ulva australis* extract 500 mg/kg (n = 5).

The purchased six-week-old male ICR mice were raised under specific pathogen-free conditions (KOSA BIO Inc., Sungnam, Republic of Korea). The mice were maintained in a stable breeding environment with a light/dark cycle of 12 h a day at a temperature of 23 ± 3 °C, relative humidity of 50 ± 10%, and 150 to 300 Lux. The experimental animals were acclimated to the environment for one week before the experiment and allowed free access to standard feed (Samtako Bio Korea, Seoul, Republic of Korea) and water. After the adaptation period, just before administration, streptozotocin (STZ, Sigma-Aldrich) was dissolved in cold 0.09 M citrate buffer (Sigma-Aldrich) and intraperitoneally administered to the animals at 40 mg/kg for 5 days [33,34]. To confirm whether hyperglycemia was induced 72 h after the injection, blood glucose levels were measured in the tail vein using a glucometer. Animals with blood glucose levels of 250 mg/dL were selected for this study. The ICR mice were divided into six groups: (1) normal, (2) diabetic, (3) metformin (100 mg/kg/day), (4) SH (500 mg/kg/day), and (5) UA (500 mg/kg/day) (Scheme 1) [20,35]. The groups were fed the same diet and divided into six sub-groups of five animals each. The diabetic group received PBS by oral administration, and the experimental group received metformin or its extract by oral administration for 6 weeks. This study was approved by the Institutional Animal Care and Use Committee of Catholic University of Pusan (CUP 2022-003).

### 2.10. Weekly Body Weight and Blood Glucose Analysis

To analyze the changes in the weight of the experimental animals, they were weighed using an electronic balance once a week at a fixed time (10:00 a.m.). Body weight gain was expressed as a percentage by comparing the weight measured at the end of the experiment with that measured at the beginning.

Changes in blood glucose levels were assessed every 10 days using a glucometer. The diet was restricted for 6 h before blood glucose measurement, and tail vein blood was collected for subsequent analyses.

### 2.11. Insulin Tolerance Test and Oral Glucose Tolerance Test

The insulin tolerance test (ITT) was performed on mice that fasted for 6 h, and 0.75 units/kg of insulin (Sigma-Aldrich) was intraperitoneally injected. Blood glucose levels were measured before injection and 30, 60, 90, and 120 min after injection. The area under the curve (AUC) was calculated to analyze insulin sensitivity.

An oral glucose tolerance test (OGTT) was performed after all animals had fasted for 12 h. Blood was collected from the tail vein of the experimental animals at 0, 30, 60, 90, and 120 min after oral administration of 2 g/kg glucose solution, and changes in blood glucose were analyzed.

### 2.12. Serum Biochemical Analysis

The experimental animals were fasted for 12 h before autopsy, and a mixture of 25 mg/kg alfaxalone (Alfaxan^®^; Careside, Subiaco, WA, Australia) and 5 mg/kg xylazine (Rompun^®^; Bayer Korea, Seoul, Republic of Korea) was used as an anesthetic. Blood was collected from the hearts of anesthetized experimental animals, coagulated at room temperature, and analyzed using plasma obtained by centrifugation at 2500 rpm for 30 min. The parameters analyzed were aspartate aminotransferase (AST), alanine aminotransferase (ALT), alkaline phosphatase (ALP), total protein (TP), triglycerides (TG), total cholesterol (CHO), high-density lipoprotein cholesterol (HDL-C), glucose (GLU), blood urea nitrogen (BUN), and creatinine (CRE). Analysis was performed using a blood biochemistry analyzer BT1500 (Biotecnica Instrument SpA, Rome, Italy).

### 2.13. Organ Weight and Histopathological Examination

The liver, kidney, pancreas, spleen, and heart of the experimental animals were removed and weighed, and the absolute organ weight was compared to the body weight of the experimental animals. For the histopathological examination, the liver and pancreas were partially resected and fixed in 10% neutral formalin, and paraffin blocks were prepared. After that, slices 3 μm thick were cut to prepare serial sections, which were then deparaffinized, rehydrated by gradually passing ethanol, washed, stained with hematoxylin and eosin, and finally evaluated using a microscope (Olympus BX51, Olympus Optical Co., Rome, Italy).

### 2.14. Statistical Analysis

GraphPad Prism program 5.0 software (GraphPad Software Inc., SanDiego, CA, USA) was used for data analysis. Values were expressed as mean ± standard deviation and tested with one-way ANOVA for significant differences. The significance level was set at *p* < 0.05.

## 3. Results

### 3.1. α-Glucosidase Inhibitory Activity of SH and UA

**Figure 1:**
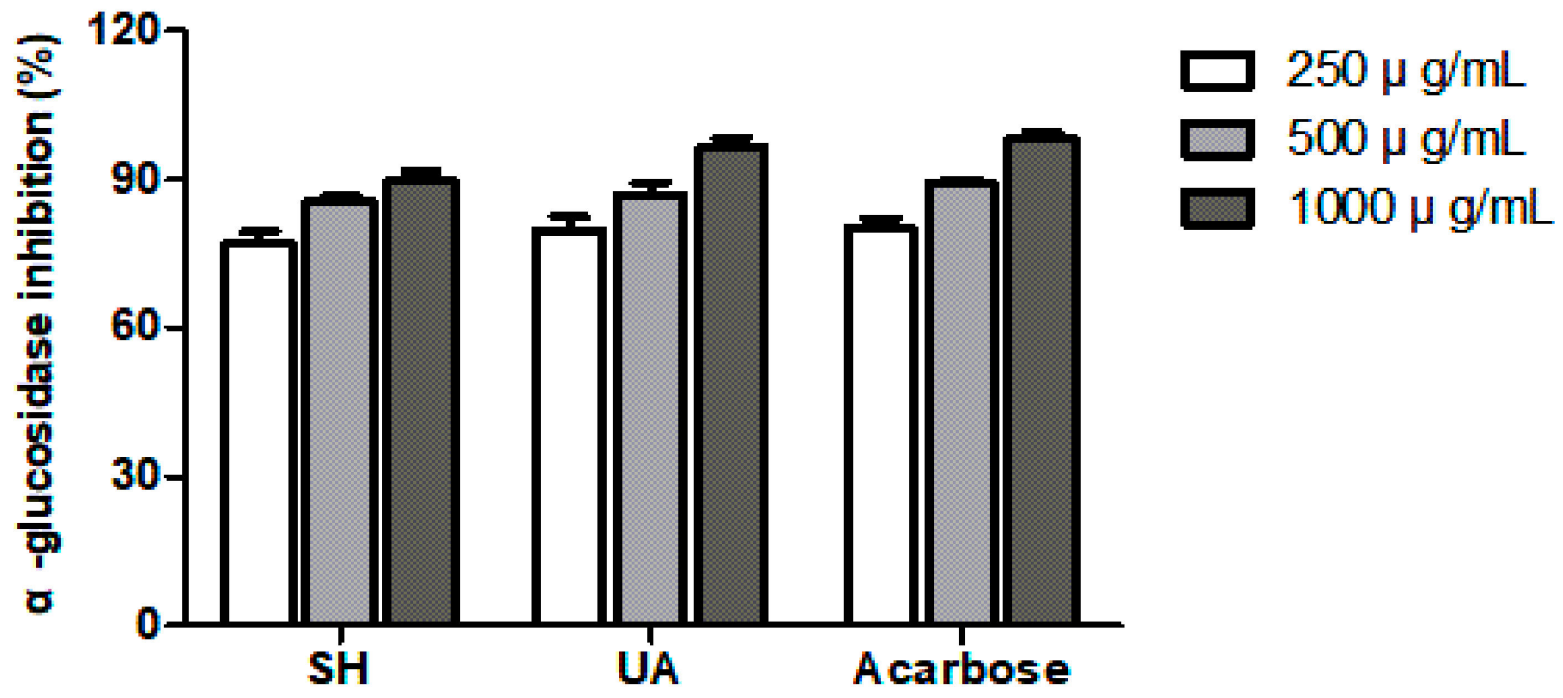
α-glucosidase inhibitory activity of *Sargassum horneri* and *Ulva australis* extracts. Acarbose was used as a positive control in the glucosidase inhibition assay. *S. horneri* is denoted as SH, and *U. australis* is denoted as UA. *U. australis* showed higher glucosidase inhibitory activity than *S. horneri*. Data are expressed as mean ± standard deviation (n = 3).

To analyze the anti-diabetic effects of SH and UA ethanol extracts, their glucosidase inhibitory abilities were evaluated. The SH extract showed α-glucosidase inhibitory activity of 77.1 ± 1.9, 85.7 ± 0.8, and 89.6 ± 1.7% in concentrations of 250, 500, and 1000 μg/mL, respectively. In the case of the UA extract, each treatment concentration showed 79.8 ± 2.2, 86.9 ± 1.9, and 96.3 ± 1.6% of α-glucosidase inhibitory activity. Acarbose, a positive control, showed 80.0 ± 1.8, 88.9 ± 1.0, and 98.2 ± 1.2% of inhibitory activity at each treatment concentration, respectively. Both extracts showed α-glucosidase inhibitory activity in a dose-dependent manner, and the α-glucosidase inhibitory activity of UA extract was similar to that of acarbose (Figure 1).

### 3.2. Cell Viability of HepG2 and IR-HepG2

**Figure 2:**
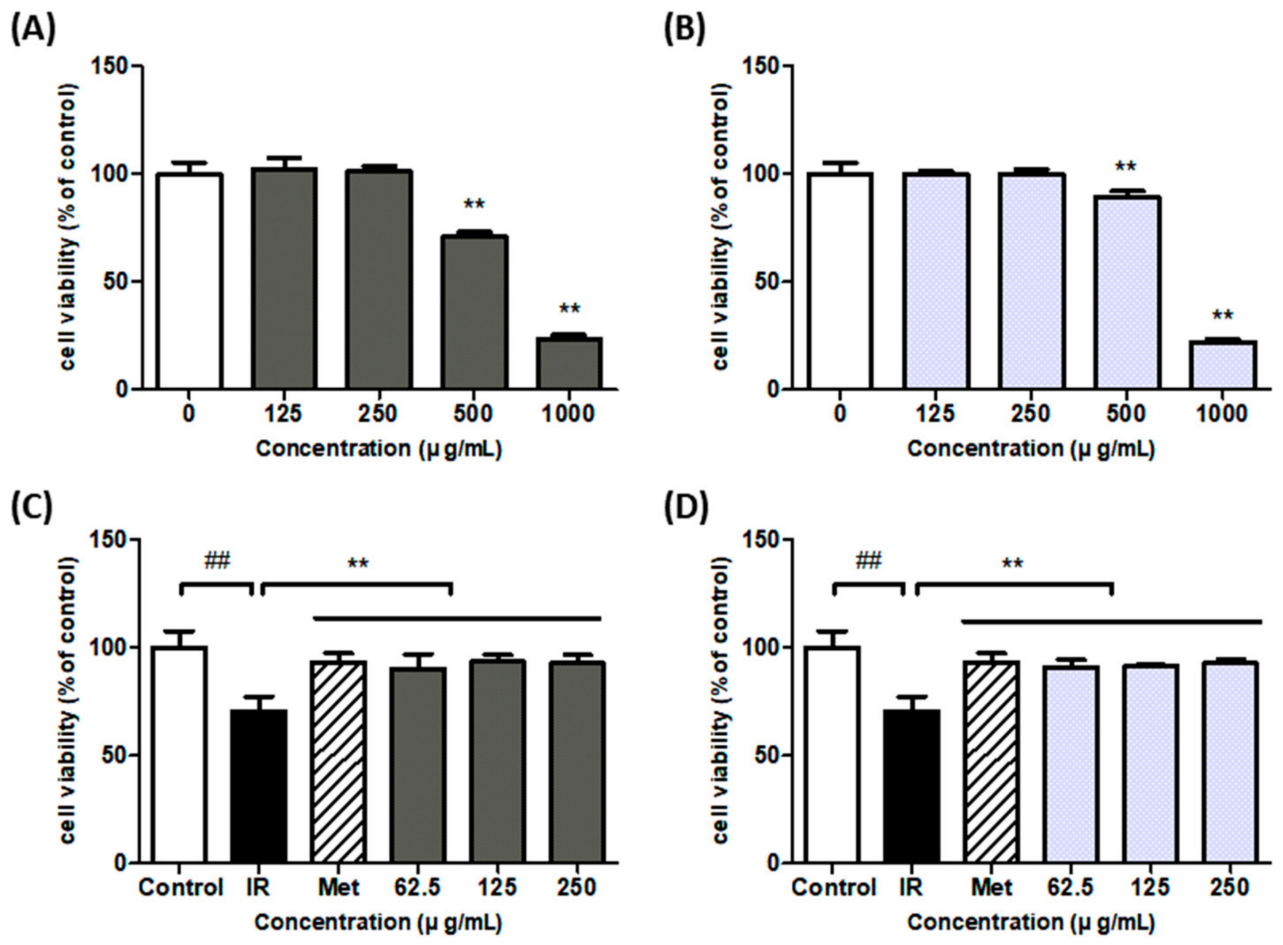
Cell viability of HepG2 and IR-HepG2 treated with *Sargassum horneri* and *Ulva australis* extracts. Evaluation of HepG2 cell viability after treatment with (**A**) *S. horneri* and (**B**) *U. australis* ethanol extracts. Significant differences: ** *p* < 0.01 vs. the control group. Evaluation of IR-HepG2 cell viability after treatment with (**C**) *S. horneri* and (**D**) *U. australis* ethanol extracts. Cell viability was measured using a WST-1 assay. Data are expressed as mean ± standard deviation (n = 3). Significant differences: ** *p* < 0.01 vs. IR group; ## *p* < 0.01 vs. NC group.

To confirm the cytotoxic effects of the SH and UA extracts, HepG2 cells were treated with different concentrations of the extract (125, 250, 500, and 1000 μg/mL). The results of the cell viability assay after 24 h of treatment showed 100% viability of cells treated with the extract at concentrations of 250 μg/mL or lower (Figure 2A,B). Thus, the concentrations used in the present study did not cause cytotoxicity.

After induction of insulin-resistant HepG2 (IR-HepG2) cells, cell viability was confirmed by treatment with the extract and metformin. The results showed that cells treated with the extract at a concentration of 250 μg/mL or lower and the positive control group treated with 1 mM metformin demonstrated a viability of approximately 90%, which was higher than that of IR-HepG2 cells (Figure 2C,D).

### 3.3. Effect of Extract Treatment on ROS Content

**Figure 3:**
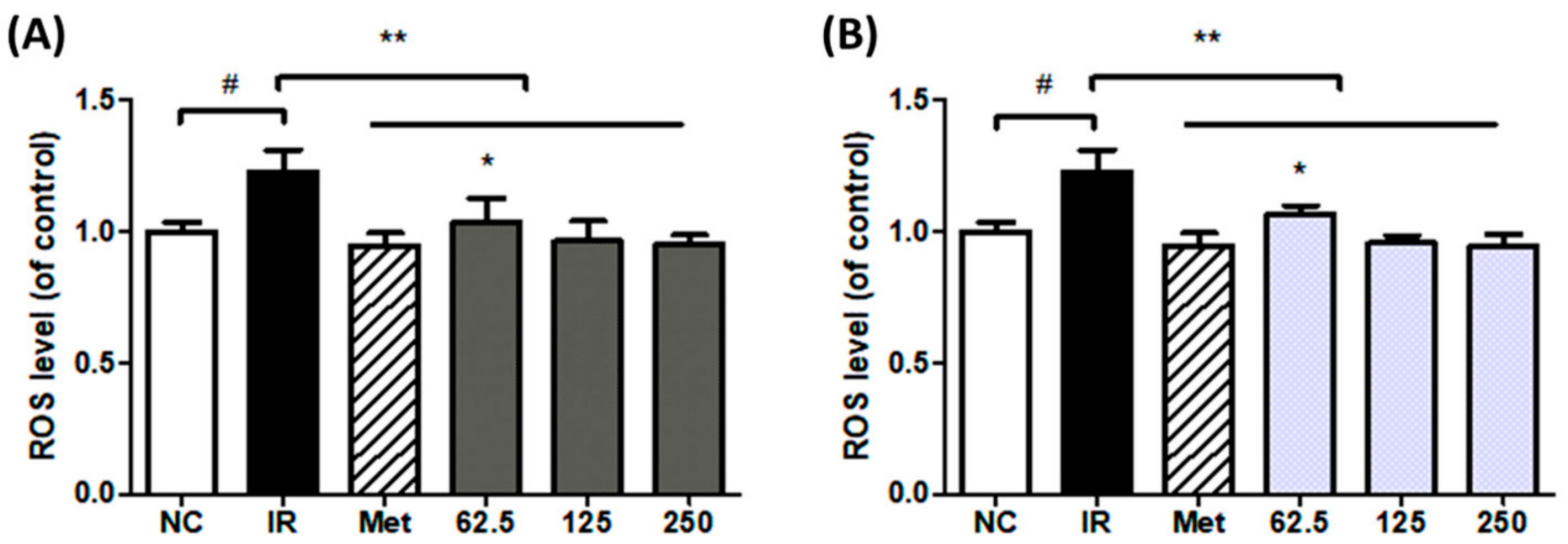
Effects of *Sargassum horneri* and *Ulva australis* treatment on the intracellular ROS content in IR-HepG2 cells. ROS content in IR-HepG2 cells was detected using a fluorescence microplate. ROS content after treatment with (**A**) *S. horneri* and (**B**) *U. australis* extracts. The ROS content in the IR-HepG2 group was significantly higher than that in the control group. Treatment with the extract and metformin significantly lowered ROS levels. Data are expressed as mean ± standard deviation (n = 3). Significant differences: * *p* < 0.05, ** *p* < 0.01 vs. IR group; # *p* < 0.05 vs. NC group.

Because IR and hyperglycemia are known to increase cellular ROS levels due to oxidative stress, we analyzed the intracellular ROS content. After preparing IR-HepG2 cells, SH and UA extracts were administered for 24 h at concentrations that did not exhibit cytotoxicity, and the ROS content was analyzed. The results revealed that the ROS content of IR-HepG2 cells increased by approximately 22% compared to that of normal cells, and the ROS levels of the experimental group treated with the extract decreased in a concentration-dependent manner. The experimental group treated with 250 μg/mL extract had ROS content similar to that of the positive control group treated with 1 mM metformin (Figure 3).

**Figure 4:**
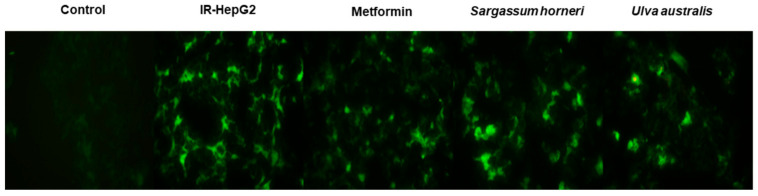
Analysis of ROS level of IR-HepG2 cells according to *Sargassum horneri* and *Ulva australis* extracts treatment using fluorescence microscopy. After the induction of IR-HepG2, the extract (250 μg/mL) and metformin (1 mM) were added and cultured for 24 h, and then the cells were stained using 10 μM DCFH-DA. When the fluorescence level of the cells was confirmed using a fluorescence microscope, IR-HepG2 cells showed overall fluorescence, whereas the fluorescence expression of the extract and metformin decreased (magnification ×400).

ROS fluorescence was confirmed using a fluorescence microscope, and the fluorescence level of IR-HepG2 cells was stronger than that of the extract- and metformin-treated groups (Figure 4). These results indicated that the SH and UA extracts reduced oxidative stress in IR-HepG2 cells.

### 3.4. Effect of Seaweed Extract on Glucose Uptake in IR-HepG2 Cells

**Figure 5:**
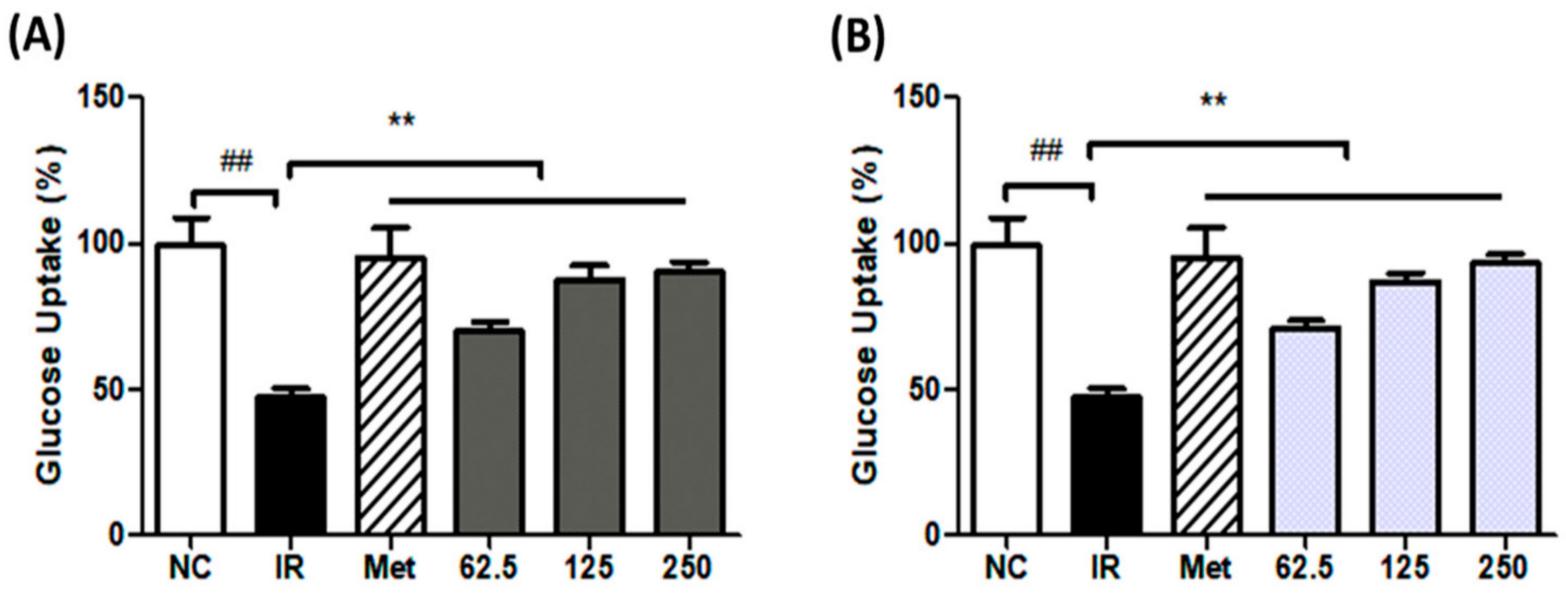
Effects of *Sargassum horneri* and *Ulva australis* treatment on glucose uptake in IR-HepG2 cells. Percentage of cellular glucose uptake (%) after (**A**) *S. horneri* extract and (**B**) *U. australis* extract treatment. The glucose uptake rate of the IR-HepG2 group was significantly lower than that of the NC group, and the glucose uptake rate significantly increased with extract and metformin treatment. Data are expressed as mean ± standard deviation (n = 3). Significant differences: ** *p* < 0.01 vs. IR group; ## *p* < 0.01 vs. NC group.

After inducing insulin resistance in HepG2 cells, the extract and metformin were administered for 24 h and glucose uptake was analyzed using 2-NBDG. The glucose uptake in IR-HepG2 cells was reduced by approximately 50% compared with that in normal cells. Treatment with SH extract at 62.5, 125, and 250 μg/mL led to glucose uptake rates of 69.7%, 87.4%, and 90.2%, respectively. The UA extract concentrations were 70.7, 86.9, and 93.2%, respectively. The positive control group treated with 1 mM metformin showed 94.9% glucose uptake. Both the extracts increased the glucose uptake rate in a concentration-dependent manner, and UA extract treatment led to a glucose uptake rate similar to that of metformin (Figure 5).

**Figure 6:**
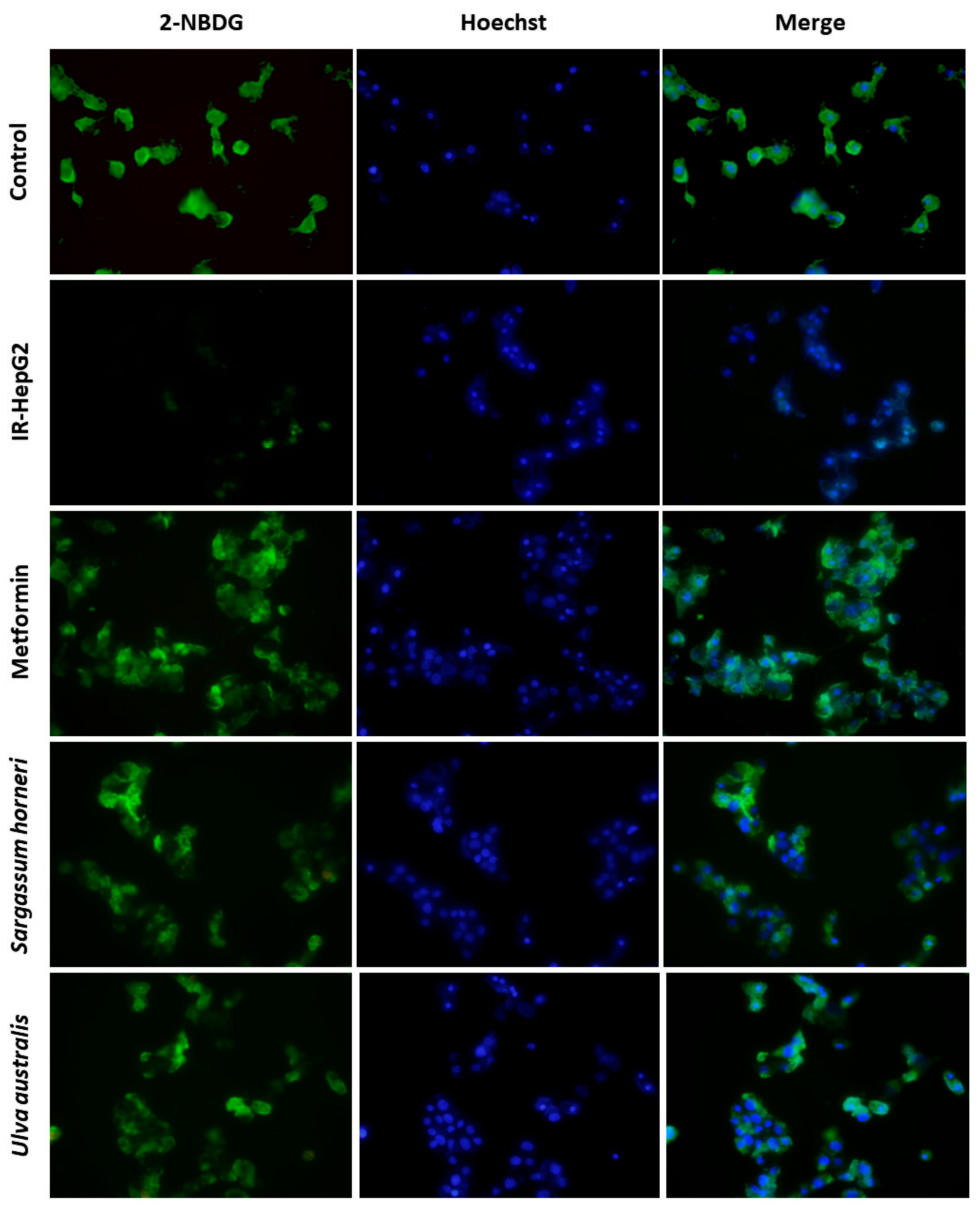
Cellular glucose uptake rate in IR-HepG2 cells with *Sargassum horneri* and *Ulva australis* extracts treatment. After induction of IR-HepG2, the extract (250 μg/mL) and metformin (1 mM) were added and cultured for 24 h. The cells were then stained with 40 μM 2-NBDG, and the fluorescence intensity of 2-NBDG was used to determine the extent of glucose uptake in control and insulin-resistant HepG2 cells. The number of cells was evaluated using Hoechst 33342 staining and confirmed using a fluorescence microscope (magnification ×400). Fluorescence intensity images were merged using ImageJ software V1.52.

After 2-NBDG staining to confirm the glucose uptake rate, and Hoechst 33342 staining to confirm the number of cells, the degree of fluorescence was evaluated using a fluorescence microscope. The results confirmed that the fluorescence intensity of the IR-HepG2 group was lower than that of the extract- and metformin-treated groups (Figure 6). These results indicated that SH and UA help mitigate the glucose uptake ability of the cells.

### 3.5. Glycogen Content of IR-HepG2 Cells According to Extract Treatment

**Figure 7:**
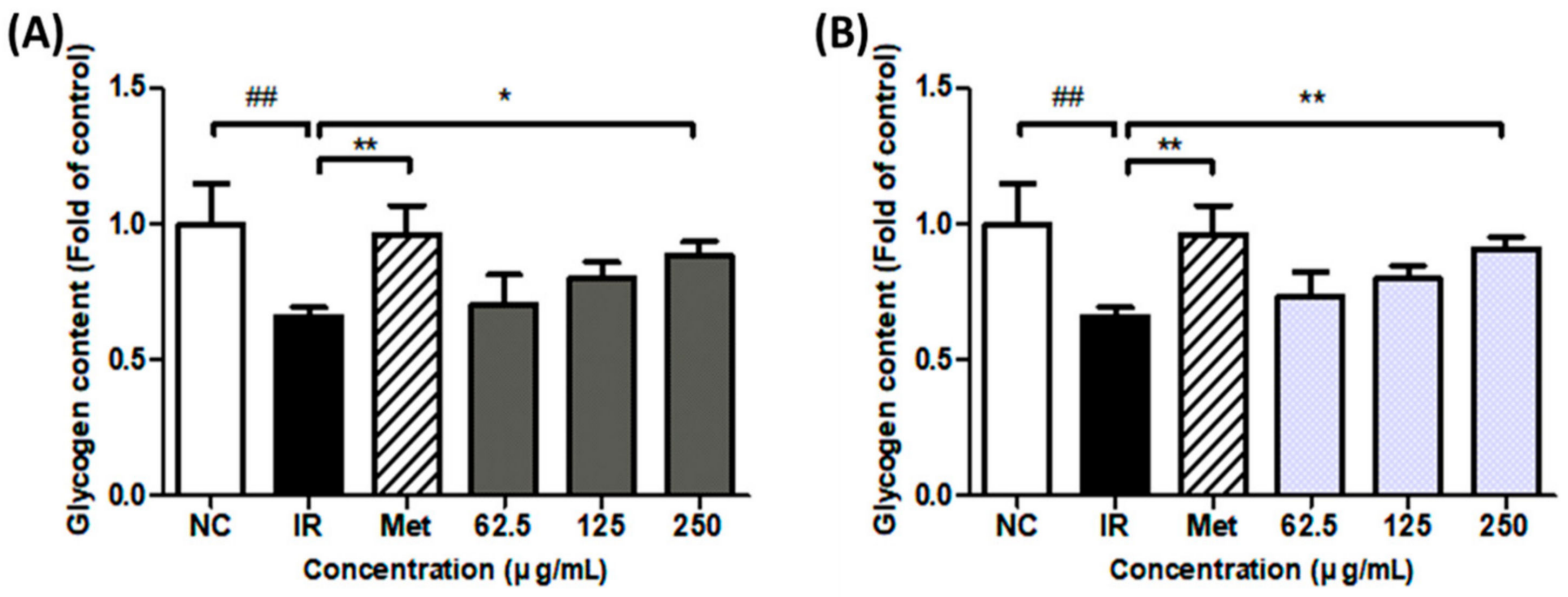
Effect of *Sargassum horneri* and *Ulva australis* on glycogen contents in IR-HepG2 cells. Intracellular glycogen content of IR-HepG2 cells following treatment with (**A**) *S. horneri* extract and (**B**) *U. australis* extract. The IR-HepG2 group exhibited significantly lower levels than the control group. Compared to the IR-HepG2 group, metformin and the extract at 1 mM were significantly higher at a concentration of 250 μg/mL. Data are expressed as mean ± standard deviation (n = 3). Significant differences: * *p* < 0.05, ** *p* < 0.01 vs. IR group; ## *p* < 0.01 vs. NC group.

HepG2 cells were treated with the extract and metformin for 24 h after inducing IR, and the glycogen content was analyzed. The glycogen content of IR-HepG2 cells was reduced by approximately 40% compared to that of normal cells. Positive controls treated with 1 mM metformin showed glycogen levels similar to normal cells. At the same time, treatment with SH and UA extracts at concentrations of 62.5, 125, and 250 μg/mL increased the glycogen content in a concentration-dependent manner (Figure 7).

### 3.6. Effect of Extract Treatment on IRS-1/AKT/GLUT4 Gene Expression

**Figure 8:**
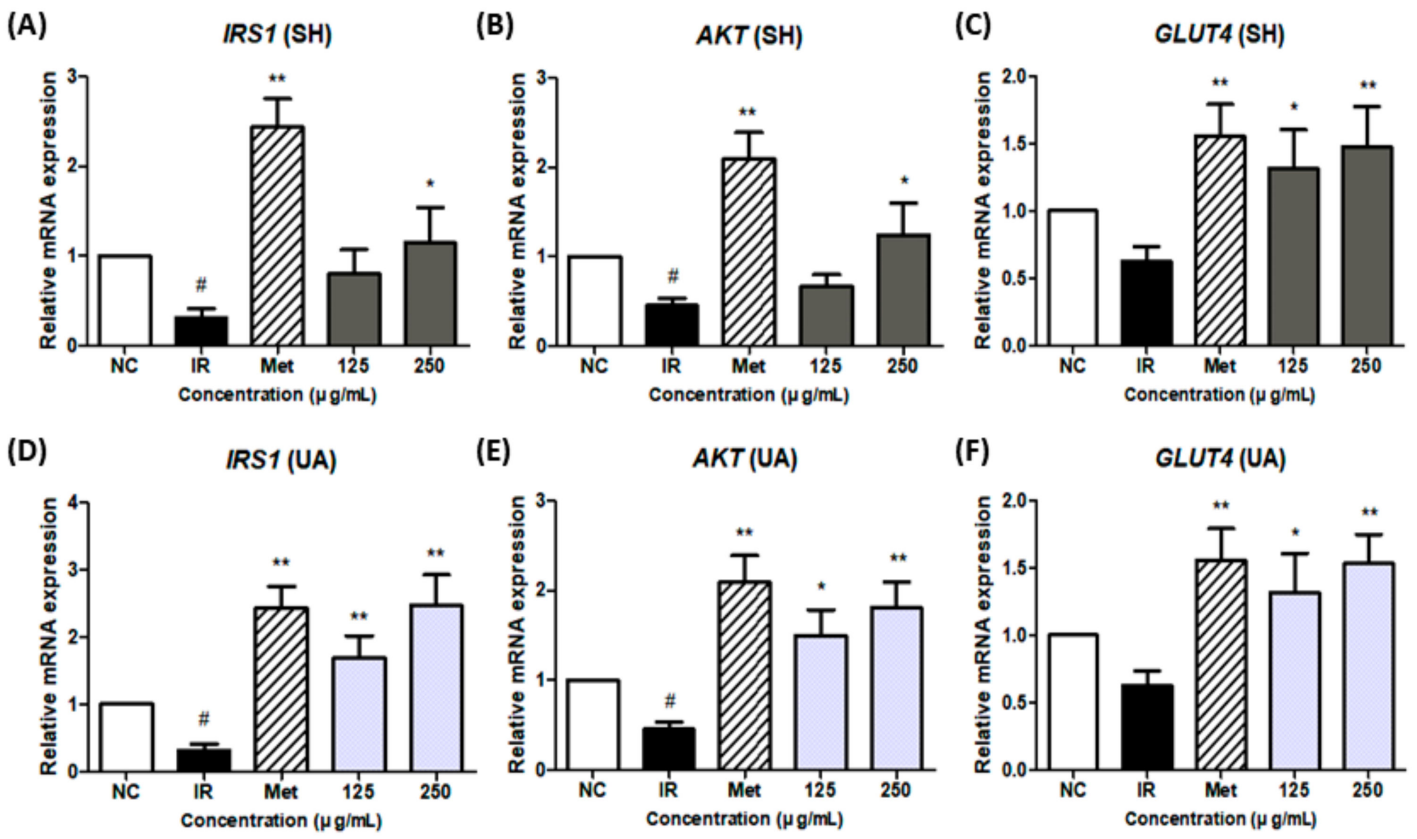
Effects of *Sargassum horneri* and *Ulva australis* on the expression of *IRS-1*/*AKT* and *GLUT4* in IR-HepG2 cells. (**A**–**C**) *IRS-1*, *AKT*, and *GLUT4* gene expression analysis according to *S. horneri* extract treatment; (**D**–**F**) *IRS-1*, *AKT*, and *GLUT4* gene expression analysis by *U. australis* extract treatment. The mRNA expression level was measured by qRT-PCR and normalized using the housekeeping gene *β-actin*. Data are expressed as mean ± standard deviation (n = 3). Significant differences: * *p* < 0.05, ** *p* < 0.01 vs. IR group; # *p* < 0.05 vs. NC group.

To investigate whether insulin resistance could be alleviated by SH and UA extracts, the expression levels of *IRS-1*/*AKT*/*GLUT4* transcription factors were measured. IR-HepG2 cells were treated with SH and UA extracts (125 and 250 μg/mL, respectively) and metformin (1 mM), which served as a positive control, and cultured for 24 h for subsequent analysis. The mRNA expression levels of *IRS-1* and *AKT* were downregulated in IR-HepG2 cells compared with those in normal cells. In the extract and metformin treatment groups, the mRNA levels were upregulated, and the results confirmed that the mRNA level in the UA extract treatment group was higher than that in the SH extract treatment group (Figure 8).

In addition, we analyzed the mRNA expression levels of the *GLUT4* gene found in skeletal muscle, hepatocytes, and adipocytes. The expression level of the *GLUT4* gene decreased in IR-HepG2 cells, and the mRNA level significantly increased in the extract- and metformin-treated groups (Figure 8C,F).

### 3.7. Effect of Glycogen-Metabolism-Related Gene Expression by Extract Treatment

**Figure 9:**
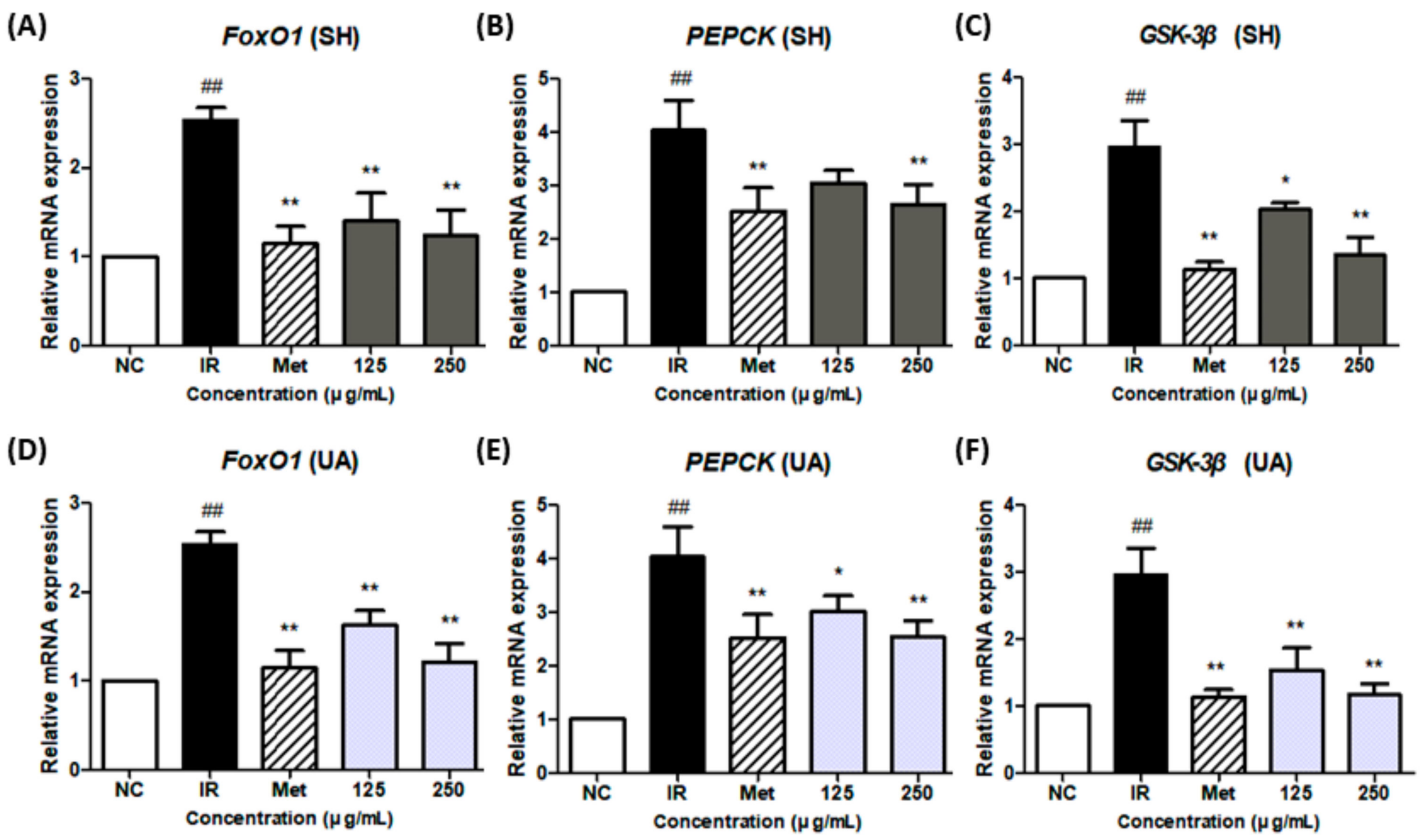
Effects of *Sargassum horneri* and *Ulva australis* on the expression of *FoxO1*, *PEPCK*, and *GSK-3β* in IR-HepG2 cells. (**A**–**C**) *FoxO1*, *PEPCK*, and *GSK-3β* gene expression analysis according to *S. horneri* extract treatment; (**D**–**F**) *FoxO1*, *PEPCK*, and *GSK-3β* gene expression analysis by *U. australis* extract treatment. The mRNA expression level was measured by qRT-PCR and normalized using the housekeeping gene *β-actin*. Data are expressed as mean ± standard deviation (n = 3). Significant differences: * *p* < 0.05, ** *p* < 0.01 vs. IR group; ## *p* < 0.01 vs. NC group.

To confirm the regulation of glycogen metabolism in IR-HepG2 cells following treatment with SH and UA extracts, the expression of *FoxO1*, *PEPCK*, and *GSK-3β* genes was investigated. The mRNA levels of *FoxO1* and *PEPCK*, which are gluconeogenesis genes regulated by *AKT*, were examined. The expression levels of *FoxO1* and *PEPCK* genes in IR-HepG2 cells were higher than those in normal cells, and, after treatment with the extract and metformin, mRNA levels significantly decreased compared to IR-HepG2 cells (Figure 9).

In addition, the mRNA expression of *GSK-3β*, which regulates glycogen synthesis in response to insulin, was analyzed. IR-HepG2 cells showed upregulated mRNA expression compared to normal cells, whereas these changes were suppressed in the extract- and metformin-treated groups (Figure 9C,F). These results confirmed that the mRNA levels of genes related to glucose metabolism in the groups treated with the SH and UA extracts were suppressed to a level similar to that in the group treated with metformin.

### 3.8. Changes in Body Weights

**Figure 10:**
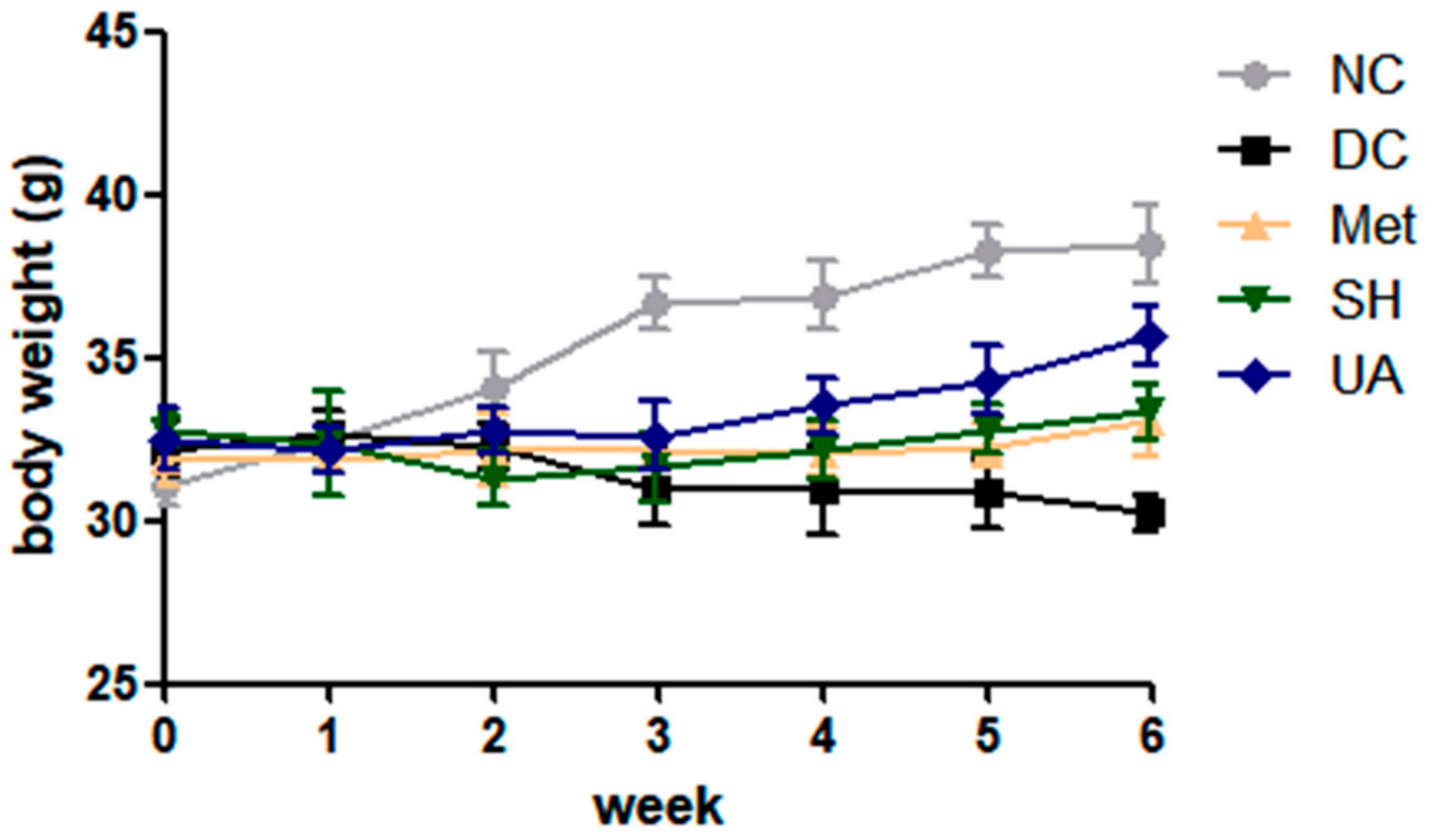
Changes in body weight for 6 weeks in diabetic mice induced by streptozotocin following oral administration of the extracts. Weekly changes in body weight. NC, normal control; DC, diabetic control; Met, metformin 100 mg/kg; SH, *Sargassum horneri* extract 500 mg/kg; UA, *Ulva australis* extract 500 mg/kg. Each value represents the mean ± standard deviation (n = 5).

The effects of the SH and UA extracts on body weight changes in animals with diabetes induced by the administration of streptozotocin (40 mg/kg) for 5 days were analyzed. Before the start of the experiment, the body weight was 31.0 to 32.5 g, and there was no difference in body weights between the experimental groups. Body weight was analyzed for six weeks after the substance was administered, and the weight gain rate of the diabetic animals was found to be lower than that of the normal group. In the group administered metformin and the extract, weight increased from the third week, but, in the diabetes group, weight gradually decreased (Figure 10).

The body weight gain was 6.93 ± 1.22 g, −0.86 ± 0.57 g, 1.28 ± 0.45 g, 1.12 ± 0.36 g, and 2.54 ± 0.43 g in the normal, diabetic, metformin-, SH-, and UA-treated groups, respectively. The group administered metformin and the extract showed significantly higher weight gain than the diabetic group, with the highest weight gain observed in the UA-treated group (Table 2). Food intake was approximately 4 g per animal in all groups tested, and no differences were found between groups. Therefore, the weight regain in animals treated orally with metformin, SH, and UA is likely due to substance administration.

### 3.9. Changes in Blood Glucose at 10-Day Intervals According to the Administration of the Extract

Blood was collected from the tail and a blood glucose analyzer was used to analyze the changes in blood glucose levels during the oral administration of SH and UA extracts for 6 weeks. The blood glucose level in the control group was 160 mg/dL. In contrast, in animals with STZ-induced diabetes, the blood sugar level was 430–440 mg/dL, and there was no difference between the experimental groups. The results of blood glucose levels 10 days after administration of the extract showed that the blood glucose level increased to 516.8 ± 43.3 mg/dL in the diabetic group. In the metformin-, SH-, and UA-treated groups, blood glucose levels decreased by 422.4 ± 43.6 mg/dL, 424.0 ± 37.6 mg/dL, and 423.0 ± 41.3 mg/dL, respectively. Subsequently, it was confirmed that blood glucose was significantly reduced in the metformin-, SH-, and UA-administered groups compared to the diabetic group (Table 3).

### 3.10. Analysis of Insulin Tolerance Test and Oral Glucose Tolerance Test

**Figure 11:**
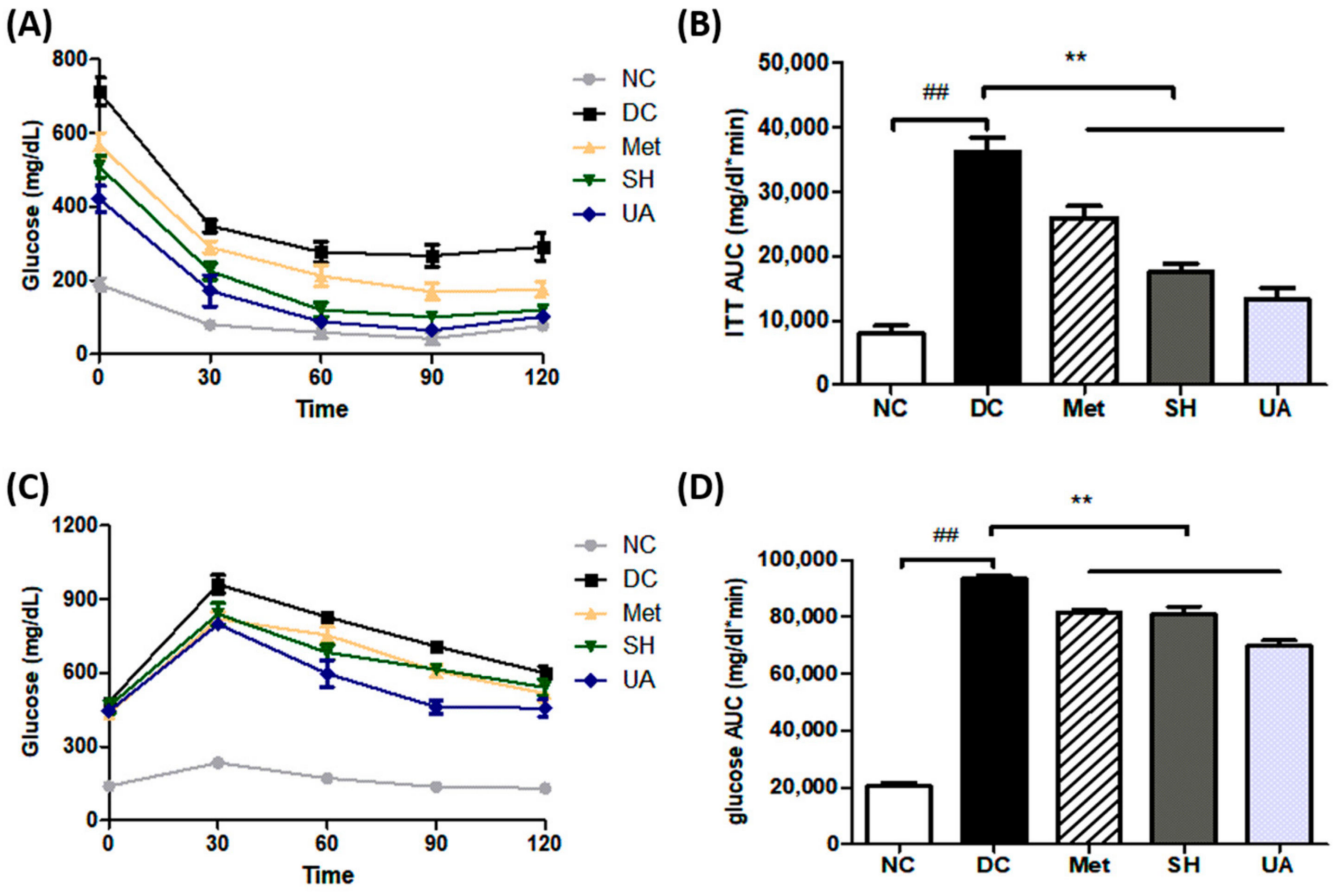
Analysis of insulin and oral glucose tolerance test in STZ-diabetic mice following the administration of the extracts. (**A**) insulin tolerance test (ITT), (**B**) area under the curve (AUC) of ITT, (**C**) oral glucose tolerance test (OGTT), and (**D**) area under the curve (AUC) of OGTT. NC, normal control; DC, diabetic control; Met, metformin 100mg/kg treatment; SH, *Sargassum horneri* extract 500mg/kg treatment; UA, *Ulva australis* extract 500 mg/kg treatment. Each value represents the mean ± standard deviation (n = 5). Significant differences: ** *p* < 0.01 vs. DC group; ## *p* < 0.01 vs. NC group.

Blood glucose levels measured 30 min after insulin injection were higher in the diabetic and drug-administered groups than in the normal group; however, insulin resistance in the drug-administered groups tended to improve compared to that in the diabetic group. The area under the curve (AUC) was determined using an insulin tolerance test. The diabetic group showed a significantly higher AUC than the normal group, whereas the metformin-, SH-, and UA-treated groups showed significantly lower AUC values than the diabetic group (Figure 11A,B).

The blood glucose levels 30 min after glucose administration were 900 mg/dL in the diabetic group and 800 mg/dL in the groups administered metformin, SH, and UA extracts. After 60 min, the blood glucose levels were the lowest in the UA-administered group. Analysis of the AUC using the oral glucose tolerance graph confirmed that the group treated with metformin, SH, and UA extracts showed significantly decreased glucose tolerance compared with the diabetic group (Figure 11C,D).

### 3.11. Serum Biochemical Analysis

The AST and ALT levels were significantly higher in all groups than in the normal group. However, the ALT levels in the UA-administered group were significantly lower than those in the diabetic group. In the case of ALP and TP levels, there were no significant differences in the values of the normal group among the experimental groups with diabetes.

The TG levels were significantly higher in all groups than in the normal group. The corresponding values amounted to 258.4 ± 26.0 mg/dL, 78.4 ± 17.3 mg/dL, 78.9 ± 16.5 mg/dL, and 79.1 ± 12.3 mg/dL in the diabetes group, metformin-, SH-, and UA-treated groups, respectively. Compared with the diabetic group, the levels were significantly lower in the groups administered metformin or the extract. Total cholesterol significantly decreased in all substance-administered groups compared with that in the diabetic group. HDL cholesterol levels were significantly higher in the metformin- and UA-treated groups than in the diabetic group.

Blood glucose levels were significantly higher in animals inoculated with streptozotocin compared to those in the normal group (98.0 ± 11.2 mg/dL). The diabetic group demonstrated blood glucose levels of 748.0 ± 38.8 mg/dL, which were significantly higher than those in the groups administered metformin or seaweed extracts. In addition, we found no significant differences in the BUN and creatinine levels between the experimental groups (Table 4).

### 3.12. Tissue Weight and Liver Index Analysis

At the end of the experiment, weights of the liver, kidneys, pancreas, spleen, and heart were measured and compared. The weight of the liver significantly increased by 2.04 ± 0.24 g in the diabetic group compared to 1.34 ± 0.08 g in the normal group. The weights of the liver in the metformin-, SH-, and UA-treated groups were 1.69 ± 0.09 g, 1.72 ± 0.08 g, and 1.68 ± 0.09 g, respectively. Liver weights in the metformin and UA groups were significantly lower than those in the diabetic group. The kidney, pancreas, spleen, and heart weights were similar to those of animals in the normal group (Table 5).

**Figure 12:**
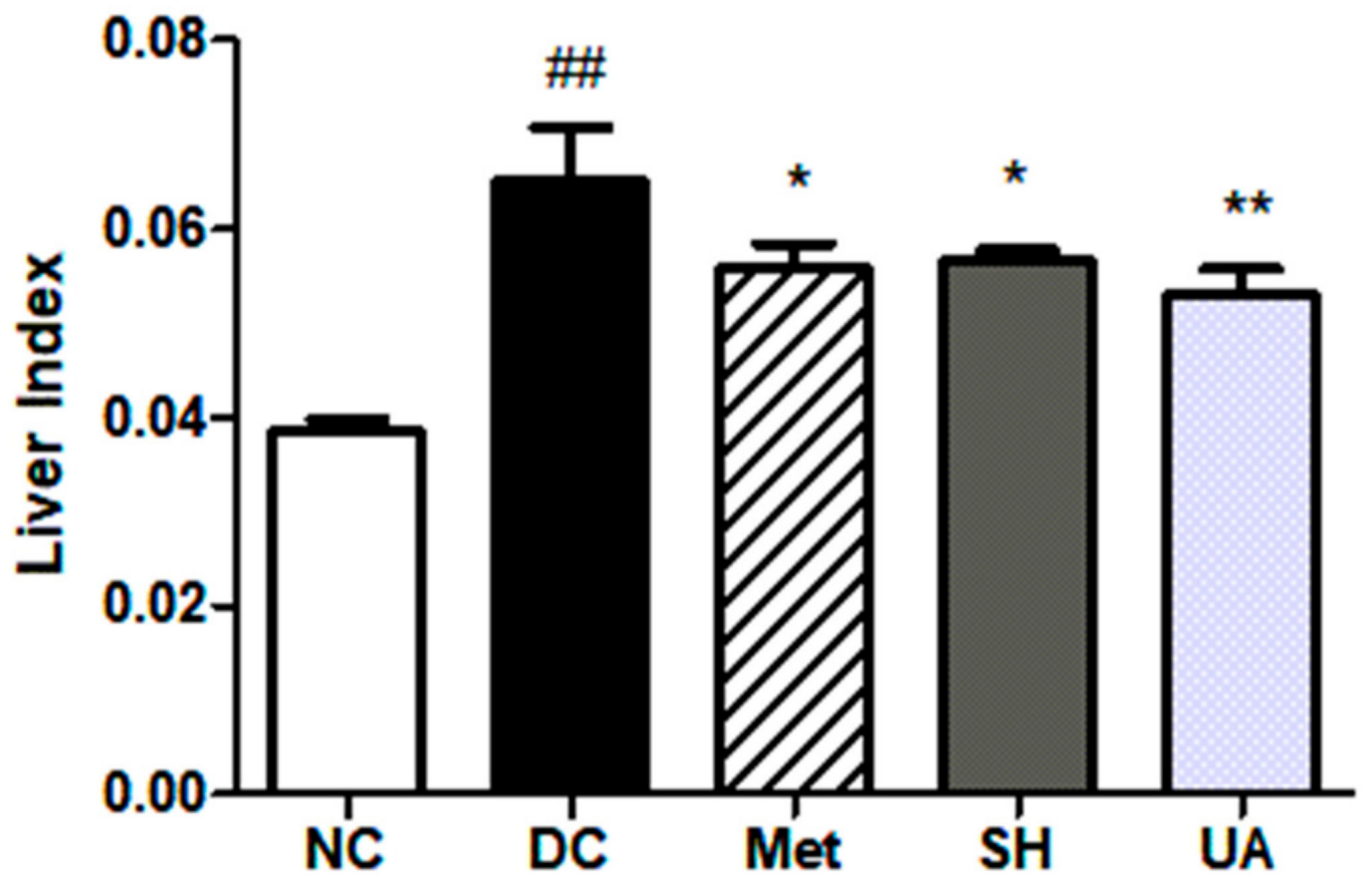
Changes in liver index in STZ-diabetic mice. Liver index after administration of *Sargassum horneri* and *Ulva australis* extracts. NC, normal control; DC, diabetic control; Met, metformin 100 mg/kg treatment; SH, *S. horneri* extract 500 mg/kg treatment; UA, *U. australis* extract 500 mg/kg treatment. Each value represents the mean ± standard deviation (n = 5). Significant differences: * *p* < 0.05, ** *p* < 0.01 vs. DC group; ## *p* < 0.01 vs. NC group.

The liver index, calculated using the body and liver weights at the end of the animal experiment, was significantly higher in the diabetic group than in the normal group. The metformin-, SH-, and UA-treated groups demonstrated significantly lower values than the diabetic group, with the UA group showing the highest significance level (Figure 12).

### 3.13. Histopathological Analysis

**Figure 13:**
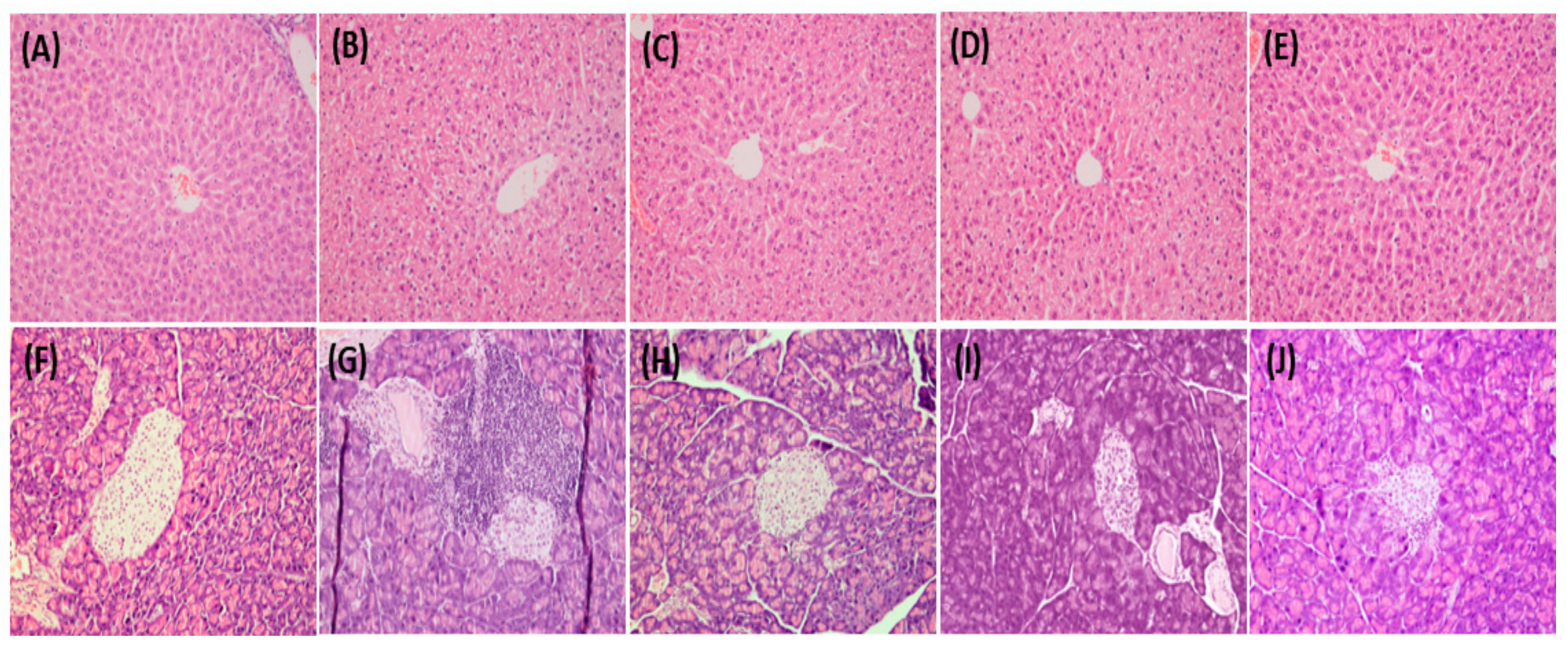
Effect of *Sargassum horneri* and *Ulva australis* on microphotographs of the liver and pancreas in STZ-diabetic mice. (**A**–**E**) Hematoxylin and eosin (**H**,**E**) stain of liver tissue; (**F**–**J**) hematoxylin and eosin (**H**,**E**) stain of pancreas tissue (magnification ×200). NC, normal control; DC, diabetic control; Met, metformin 100 mg/kg treatment; SH, *S. horneri* extract 500 mg/kg treatment; UA, *U. australis* extract 500 mg/kg treatment.

Histological analyses of liver and pancreatic tissues were performed using hematoxylin and eosin staining. In the case of the liver tissue, the lobular structure was well maintained in the normal group, and hepatocytes were formed in a single layer centered on the central vein. However, the diabetic group exhibited severe hepatocellular degeneration, including fatty acid changes and swelling. In contrast, in the group administered metformin, SH, and UA, the hepatic lobules were well maintained, and the degree of fatty degeneration was significantly reduced compared to that in the diabetic group (Figure 13A–E).

In the case of the pancreas, it was confirmed that, in the normal group, the shape of the islets of Langerhans was conspicuously well preserved, the endocrine and exocrine structures were well maintained, and acini cells were abundant. However, in the diabetic group, atrophied cells were observed, the structure of the islets of Langerhans was abnormal, and severe inflammatory cells were observed around the blood vessels and islets. In the group administered metformin, SH, and UA, the size of the islets of Langerhans tended to shrink compared to that in the normal group, but the number of inflammatory cells was lower than that in the diabetic group (Figure 13F–J).

## 4. Discussion

Diabetes is a group of chronic diseases associated with hyperglycemia and characterized by excessive concentrations of circulating glucose in the blood. Patients with diabetes are over four times more likely than healthy controls to suffer from diseases such as hypertension, vascular disease complications, blindness, and renal failure [36]. Drugs used for the treatment of diabetes include oral hypoglycemic agents and insulin; however, these drugs have been reported to cause many side effects related to the gastrointestinal system [37,38]. Accordingly, diet and medication have been reported to be the most commonly used methods to treat diabetes. For example, consuming fruits, whole grains, and vegetables can prevent or reduce type 2 diabetes [39]. In addition, research is being conducted to develop new drugs using natural products with fewer side effects to prevent and treat diabetes and to use them as dietary supplements [40].

Seaweed, one of the foods used to prevent diabetes, contains polyphenols, carotenoids, vitamins, phycobilins, and physiologically active compounds in the form of polysaccharides [41,42]. Seaweeds used as food are suitable for diabetes management because of their low-calorie content and richness in dietary fiber, unsaturated fatty acids, and vitamins [43]. However, SH and UA, which are abundant in East Asia, are not consumed domestically, and have been identified as the cause of coastal pollution on Jeju Island. In the present study, extracts were prepared from these two types of marine waste, which cause environmental problems and are generated in volumes of approximately 10,000 tons per year. Subsequently, their anti-diabetic effects were analyzed in insulin-resistant HepG2 cells and in animals with STZ-induced diabetes.

Alpha-glucosidase is an important enzyme involved in carbohydrate digestion that catalyzes the breakdown of ingested polysaccharides into monosaccharides, which are then absorbed. Inhibitors of this enzyme are important targets for the treatment of diabetes because they inhibit carbohydrate digestion and glucose absorption [44]. The results of our analysis of α-glucosidase inhibitory ability confirmed that SH and UA extract increased α-glucosidase inhibitory ability in a concentration-dependent manner. Furthermore, the UA extract exhibited an effect similar to that of acarbose, the positive control. This suggests a higher efficacy of SH compared to the 93.2% inhibitory effect of the *Sargassum fulvellum* hexane fraction at a concentration of 2 mg/mL [45]. Notably, UA showed a higher efficacy than the 88.0% inhibitory ability reported for a concentration of 4 mg/mL *Ulva lactuca* [46]. Thus, SH and UA increased α-glucosidase inhibition in vitro to levels similar to the positive control drug acarbose, but further analysis is needed to replace acarbose, which is known to cause abdominal pain in patients [47].

The liver plays a significant role in regulating blood sugar levels via glycogen synthesis and glucose metabolism [48]. HepG2 cells derived from human liver cancer tissues exhibit biological activities similar to those of normal hepatocytes and are extensively used in insulin resistance studies because exposure to insulin causes cellular damage [49]. The efficacy of SH and UA extracts was analyzed after inducing insulin resistance in HepG2 cells. In cells with induced insulin resistance, intracellular ROS levels increase significantly, thereby suppressing tissue antioxidant defenses and causing oxidative stress [50]. Therefore, ROS levels were analyzed after treatment with the two extracts. Following treatment with SH and UA extracts, the ROS levels tended to decrease in a concentration-dependent manner and were similar to the corresponding trends observed in the metformin-treated group, indicating that the extracts can help relieve oxidative stress. As insulin resistance is associated with difficulties in regulating glucose production owing to impaired glucose absorption and glycogen synthesis, the therapeutic effects of SH and UA extracts have been confirmed [51]. Glucose uptake and glycogen content decreased in insulin-resistant HepG2 cells but were alleviated in a concentration-dependent manner in the experimental group treated with the extract. This was similar to the metformin-treated positive control group, suggesting that SH and UA can help alleviate insulin resistance.

In general, the *IRS-1*/*PI3K*/*AKT* signaling pathway plays an essential role in regulating glucose metabolism, including glycogen synthesis, promotion of glucose synthesis, and inhibition of angiogenesis in the liver [52]. The activation *IRS-1* further activates other signaling pathways, such as the *PI3K*/*AKT* pathway, followed by the activation of targets such as *GLUT4*, *GSK-3β*, and *PEPCK* [53]. However, when insulin resistance is induced, the signaling of *IRS-1*, an insulin receptor substrate, and *AKT*, which regulates glucose and lipid metabolism, is downregulated, thereby affecting the expression of downstream factors. Treatment of insulin-resistant HepG2 cells with SH and UA extracts upregulated *IRS-1*/*AKT* and *GLUT4* expression. These findings are consistent with those of a previous report showing that monounsaturated fatty acids in seaweeds can help preserve the *IRS*/*PI3K* insulin pathway and increase *GLUT4* translocation [54]. In addition, *FoxO1*, a significant target of *AKT*, lowers the glucose level and induces the expression of *PEPCK* to enhance gluconeogenesis [55,56], and *GSK-3β* is known to regulate glycogen synthase, an enzyme that stores glycogen [57]. In the present study, as the expression of *AKT* was upregulated by treatment with SH and UA extracts, the gene expression of *FoxO1*, a major target, was downregulated, similar to that of *PEPCK* and *GSK-3β*. These results suggest that the two seaweed extracts inhibit or improve the expression of key transcription factors involved in insulin resistance.

Diabetic mice with STZ-induced diabetes are a model similar to human diabetes; therefore, they have been widely used to investigate antidiabetic activity [58]. Streptozotocin has been previously reported to induce diabetes by causing the death of pancreatic β cells by alkylation of DNA, reducing insulin synthesis and release [59]. In the present study, the positive control substances, metformin, SH, and UA, were orally administered for 6 weeks to confirm their antidiabetic effects on streptozotocin-induced ICR mice. As weight loss is a common symptom of diabetes, no significant weight change was observed in the experimental group with induced diabetes until the second week. Weight gain in the diabetic group decreased by 0.86 g; the metformin- and SH-treated groups showed an increase of 1.25 g and 1.12 g, respectively, while the UA group showed the highest increase rate of 2.54 g. These findings are consistent with previous findings that *Sargassum wightii* methanol extract and *Caulerpa racemose* extract prevent weight loss in diabetic animals [60,61].

Insulin resistance and oral glucose tolerance tests are routinely used to assess glycemic control in diabetic animals [62]. Insulin and oral glucose tolerance according to the administration of the extract were compared using the AUC of the blood glucose graph; the AUC of the substance-administered group was significantly lower than that of the diabetic group, indicating an improvement in glucose tolerance. Seaweeds of the genus *Sargassum* contain phlorotannins and phloroglucinol, which regulate blood glucose levels by delaying carbohydrate absorption in streptozotocin-induced diabetic mice [63]. Polysaccharides extracted from *Ulva lactuca* inhibit important enzymes involved in carbohydrate and fat metabolism in blood and the small intestine [64]. The decrease in blood glucose levels following SH and UA administration was thought to be caused by components of the seaweed.

In patients with diabetes, the accumulation of cholesterol and triglycerides is accompanied by a reduction in high-density lipoprotein cholesterol levels; these changes are major risk factors for coronary artery disease [65]. In addition, persistent hyperglycemia affects the permeability of the hepatocyte membrane, causing hepatotoxicity or glomerular filtration dysfunction; therefore, serological analysis is an important indicator [66,67]. AST and ALT levels, which are indicators of liver function, were significantly higher in all groups than in the normal group. At the same time, the ALT levels were significantly lower in the UA group compared to those in the diabetic group. These results suggest that UA alleviates chronic hyperglycemia-induced liver damage more effectively than SH. Among the blood lipid test parameters, triglyceride and cholesterol levels were significantly lower in the metformin, SH, and UA groups than in the diabetic group. HDL cholesterol levels significantly increased only in the metformin and UA groups. These results are believed to be attributable to the finding that high omega-6 fatty acids in Ulva can reduce insulin resistance through other mechanisms, including anti-inflammatory action, and triglyceride and low-density lipoprotein reduction [68]. Several studies have reported hepatomegaly due to hepatic steatosis and glycogen accumulation in patients with diabetes [69]. Comparing the changes in liver tissue weight after treatment with the two seaweed extracts, we found that hepatomegaly was significantly reduced in the UA and metformin groups compared to that in the diabetic group.

Therefore, the results of the present study confirmed that SH and UA possess alpha-glucosidase inhibitory activity in vitro, suggesting that the two seaweed extracts may be candidates to improve intracellular insulin resistance. In addition, compared with metformin, a positive control substance in diabetic animal models, both seaweeds could control blood sugar, but the effect of UA was found to be more profound. These results suggest that SH and UA, recognized as environmental pollutants in Jeju, can be used as functional additives for the treatment of diabetes and as healthy food candidates. However, in future studies, it will be necessary to investigate the active ingredients in SH and UA by analyzing fractions prepared using various organic solvents.

## Figures and Tables

**Table 1:** Primers used in quantitative real-time PCR.

Gene	GenBank Accession	Forward Primer (5′ to 3′)	Reverse Primer (5′ to 3′)	Amplicon (bp)
*IRS-1* ^1^	NM_005544.3	GAG TCC CAG CAC CAA CAG AA	CCT TGC CAC CCA TGC AGA TA	374
*AKT* ^2^	NM_005163.2	GGA CAA GGA CGG GCA CAT TA	CGA CCG CAC ATC ATC TCG TA	192
*GLUT4* ^3^	NM_001042.3	GCT GAA GGA TGA GAA GCG GA	TGT CTC GAA GAT GCT GGT CG	172
*GSK-3β* ^4^	NM_002093	CGA GAC ACA CCT GCA CTC TT	TCT GTC CAC GGT CTC CAG TA	163
*FoxO1* ^5^	NM_002015	GTG GAT GGT CAA GAG CGT GC	TGC CAC CCT CTG GAT TGA GC	170
*PEPCK* ^6^	NM-002591.3	AAG AGA CAC AGT GCC CAT CC	ACG TAG GGT GAA TCC GTC AG	201
*β-actin* ^7^	NM_001101.5	ATG GAT GAT GAT ATC GCC GCG	TCT CCA TGT CGT CCC AGT TG	250

^1^ *IRS-1*, insulin receptor substrate 1; ^2^ *AKT*, protein kinase B; ^3^ *GLUT4*, Glucose Transporter Type 4; ^4^ *GSK-3β*, glycogen synthase kinase-3 beta; ^5^ *FoxO1*, forkhead box O1; ^6^ *PEPCK*, phosphoenolpyruvate carboxykinase; ^7^ *β-actin*, homo sapiens actin beta.

**Table 2:** Changes in body weight gain of streptozotocin-induced diabetic mice following oral administration of the seaweed extract.

Groups	Initial Weight (g)	Final Weight (g)	Body Weight Gain (g)
NC	31.03 ± 0.61	38.44 ± 1.22	6.93 ± 1.22
DC	31.96 ± 0.78	30.24 ± 1.06	−0.86 ± 0.57 ^##^
Met	31.88 ± 0.79	33.07 ± 1.08	1.28 ± 0.45 *
SH	32.54 ± 0.47	33.33 ± 0.88	1.12 ± 0.36 *
UA	32.49 ± 0.95	35.65 ± 0.93	2.54 ± 0.43 **

Data are expressed as mean ± standard deviation (n = 5). Significant differences: * *p* < 0.05, ** *p* < 0.01 vs. DC group; ^##^ *p* < 0.01 vs. NC group.

**Table 3:** Changes in blood glucose at 10-day intervals in diabetic mice induced by streptozotocin following oral administration of the extracts.

Groups	Date of the Blood Glucose Measurement				
	0	10	20	30	40
NC	160.00 ± 12.01	157.50 ± 13.83	146.25 ± 9.86	128.50 ± 11.61	124.00 ± 12.71
DC	440.50 ± 48.74 ^##^	516.75 ± 43.27 ^##^	521.75 ± 33.93 ^##^	531.25 ± 55.43 ^##^	547.75 ± 36.62 ^##^
Met	437.00 ± 36.40	422.40 ± 43.63 *	414.25 ± 23.16 **	418.00 ± 43.47 *	452.25 ± 47.27 *
SH	435.50 ± 51.50	424.00 ± 37.30 *	391.50 ± 42.80 **	392.30 ± 46.80 **	446.30 ± 33.00 *
UA	434.17 ± 57.22	423.00 ± 41.32 *	359.75 ± 32.96 **	336.25 ± 29.79 **	387.00 ± 42.07 **

Data are expressed as mean ± standard deviation (n = 5). Significant differences: * *p* < 0.05, ** *p* < 0.01 vs. DC group; ^##^ *p* < 0.01 vs. NC group.

**Table 4:** Changes in serum biochemical parameters in mice with streptozotocin-induced diabetes.

	NC	DC	Met	SH	UA
AST (U/L)	131.3 ± 21.6	249.6 ± 27.0 ^##^	247.8 ± 19.8	236.5 ± 25.5	238.7 ± 22.1
ALT (U/L)	23.5 ± 3.3	64.5 ± 8.7 ^##^	62.5 ± 9.4	54.5 ± 7.8	36.8 ± 7.5 **
ALP (U/L)	93.1 ± 9.9	130.5 ± 28.0	128.3 ± 15.0	110.2 ± 16.3	111.5 ± 16.0
T-P (g/dL)	5.0 ± 0.3	4.9 ± 0.5	4.6 ± 0.5	5.0 ± 0.6	5.0 ± 0.4
TG (mg/dL)	16.1 ± 3.0	258.4 ± 26.0 ^##^	78.4 ± 17.3 **	78.9 ± 16.5 **	79.1 ± 12.3 **
CHO (mg/dL)	90.0 ± 8.6	125.5 ± 12.7 ^##^	93.5 ± 9.3 *	96.3 ± 8.7 *	97.0 ± 8.4 *
HDL-C (mg/dL)	112.5 ± 6.9	83.7 ± 7.0 ^#^	117.4 ± 14.5 *	92.6 ± 7.3	116.0 ± 12.9 *
GLU (mg/dL)	98.0 ± 11.2	748.0 ± 38.8 ^##^	624.0 ± 38.5 **	658.0 ± 38.1 **	602.0 ± 34.8 **
BUN (mg/dL)	30.5 ± 2.6	39.4 ± 8.5	28.7 ± 3.0	28.4 ± 3.4	28.2 ± 3.7
CRE (mg/dL)	0.4 ± 0.1	0.7 ± 0.2	0.5 ± 0.1	0.5 ± 0.1	0.5 ± 0.1

Data are expressed as mean ± standard deviation (n = 5). Significant differences: * *p* < 0.05, ** *p* < 0.01 vs. DC group; ^#^ *p* < 0.05, ^##^ *p* < 0.01 vs. NC group.

**Table 5:** Tissue weight changes in diabetic animals by treatment with seaweed extract.

	NC	DC	Met	SH	UA
Liver	1.34 ± 0.08	2.04 ± 0.24 ^##^	1.69 ± 0.09 *	1.72 ± 0.08	1.68 ± 0.09 *
Kidney	0.59 ± 0.05	0.58 ± 0.09	0.58 ± 0.06	0.55 ± 0.03	0.56 ± 0.03
Pancreas	0.18 ± 0.02	0.21 ± 0.05	0.20 ± 0.02	0.18 ± 0.01	0.17 ± 0.02
Spleen	0.09 ± 0.01	0.11 ± 0.02	0.08 ± 0.02	0.11 ± 0.04	0.09 ± 0.02
Heart	0.18 ± 0.02	0.16 ± 0.01	0.15 ± 0.01	0.16 ± 0.01	0.17 ± 0.01

Each value represents the mean ± standard deviation (n = 5). Significant differences: * *p* < 0.05 vs. DC group; ^##^ *p* < 0.01 vs. NC group.

## Data Availability

Not applicable.

## Abbreviations

DM:	Diabetes mellitus
IR:	insulin resistance
SH:	*Sargassum horneri*
UA:	*Ulva australis*
ROS:	Reactive oxygen species
STZ:	streptozotocin
NC:	Normal control
DC:	Diabetic control
Met:	Metformin
AUC:	area under the curve
AST:	aspartate aminotransferase
ALT:	alanine aminotransferase
ALP:	alkaline phosphatase
T-P:	total protein
TG:	triglycerides
CHO:	total cholesterol
HDL-C:	high-density lipoprotein cholesterol
GLU:	glucose
BUN:	blood urea nitrogen
CRE:	creatinine
